# The Utility of Serum IgG4 Concentrations as a Biomarker

**DOI:** 10.1155/2012/198314

**Published:** 2012-03-25

**Authors:** Shigeyuki Kawa, Tetsuya Ito, Takayuki Watanabe, Masahiro Maruyama, Hideaki Hamano, Masafumi Maruyama, Takashi Muraki, Norikazu Arakura

**Affiliations:** ^1^Center for Health, Safety, and Environmental Management, Shinshu University, 3-1-1 Asahi, Matsumoto 390-8621, Japan; ^2^Department of Gastroenterology, School of Medicine Shinshu University, 3-1-1 Asahi, Matsumoto 390-8621, Japan; ^3^Department of Medical Information, School of Medicine Shinshu University, 3-1-1 Asahi, Matsumoto 390-8621, Japan

## Abstract

IgG4-related disease is a new disease entity involving IgG4 in its clinical presentation and having 6 characteristic features: (1) systemic involvement; (2) solitary or multiple lesions showing diffuse or localized swelling, masses, nodules, and/or wall thickening on imaging; (3) high serum IgG4 concentration >135 mg/dL; (4) abundant infiltration of lymphoplasmacytes and IgG4-bearing plasma cells; (5) a positive response to corticosteroid therapy; and (6) complications of other IgG4-related diseases. To date, most IgG4-related diseases have been recognized as extrapancreatic lesions of autoimmune pancreatitis. This paper will discuss the utility of IgG4 as a biomarker of IgG4-related diseases, including in the diagnosis of autoimmune pancreatitis and its differentiation from pancreatic cancer, in the prediction of relapse, in the long-term follow-up of patients with autoimmune pancreatitis and normal or elevated IgG4 concentrations, and in patients with autoimmune pancreatitis and extrapancreatic lesions, as well as the role of IgG4 in the pathogenesis of IgG4-related disease.

## 1. Introduction

 IgG4-related disease is a new disease entity involving IgG4 in its clinical presentation. To date, 6 characteristic features of IgG4-related disease have been identified: (1) systemic involvement; (2) solitary or multiple lesions showing diffuse or localized swelling, masses, nodules, and/or wall thickening on imaging; (3) high serum IgG4 concentrations >135 mg/dL; (4) abundant infiltration of lymphoplasmacytes and IgG4-bearing plasma cells; (5) a positive response to corticosteroid therapy; (6) complications of other IgG4-related diseases [1–4].

 IgG4-related disease involves organs throughout the entire body. The major manifestations of IgG4-related disease include autoimmune pancreatitis, lacrimal and salivary gland lesions known as Mikulicz's disease, sclerosing cholangitis, retroperitoneal fibrosis, lung disease, and tubulointerstitial nephritis. In addition, many minor lesions have been reported in patients with IgG4-related disease, including hypophysitis, thyroiditis, hepatopathy, and prostatitis. At present, it is not clear whether these lesions are caused by the same etiology or merely show clinical and pathological findings associated with IgG4. Imaging modalities have shown diffuse or localized swelling in the pancreas and the lacrimal and salivary glands, masses in patients with retroperitoneal fibrosis, nodules in patients with lung pseudotumors, and wall thickening in the bronchi and bile ducts.

 Most patients with IgG4-related disease have high serum IgG4 concentrations, over 135 mg/dL, [5] a finding both sensitive and specific for this disease, as well as useful for its diagnosis. Characteristic pathological findings include the infiltration of large numbers of lymphoplasmacytes and IgG4 bearing plasma cells [6]. Although storiform or swirling fibrosis and obstructive phlebitis are also characteristics of IgG4-related disease, they are rarely observed in specific lesions, such as those of the salivary glands. Most lesions, except for those that are predominantly fibrotic, respond positively to corticosteroid therapy. For example, patients with autoimmune pancreatitis show reduced swelling, patients with lung pseudotumors show the disappearance of nodules, and patients with sclerosing cholangitis show the disappearance of bile duct strictures after corticosteroid treatment. At present, most IgG4-related diseases have been recognized as extrapancreatic lesions of autoimmune pancreatitis [2, 7, 8].

 This paper will discuss the utility of serum IgG4 concentrations as a biomarker of the major IgG4-related disease, autoimmune pancreatitis. Topics will include IgG4 concentration and the diagnosis of autoimmune pancreatitis, as well as its differentiation from pancreatic cancer; IgG4 and the prediction of relapse; long-term followup of patients with autoimmune pancreatitis and either normal or elevated IgG4 concentration; IgG4 and extrapancreatic lesions in patients with autoimmune pancreatitis; and the role of IgG4 in the pathogenesis of IgG4-related disease.

## 2. IgG4 and the Diagnosis of Autoimmune Pancreatitis

 The sera of patients with autoimmune pancreatitis have a polyclonal band in the rapidly migrating fraction of *γ*-globulins, resulting in *β*–*γ* bridging. Immunoprecipitation assays have confirmed that this band is always due to elevation of IgG4 concentration [5]. IgG is composed of 4 subclasses, IgG1, IgG2, IgG3, and IgG4. In normal subjects, IgG4 constitutes only 3–7% of total serum IgG. However, serum IgG4 concentrations are over 10-fold higher in patients with autoimmune pancreatitis. Elevated serum IgG4 has also been observed in individuals with allergic disorders, parasite infestations, and pemphigus. High serum IgG4 concentrations have been observed in 90% of patients with autoimmune pancreatitis, but rarely in patients with pancreatic cancer, chronic pancreatitis, primary biliary cirrhosis, primary sclerosing cholangitis, and Sjögren's syndrome, suggesting that IgG4 is a sensitive and specific marker of autoimmune pancreatitis and may be diagnostic for this disease [5]. Corticosteroid therapy significantly reduces serum IgG4 concentration and the IgG4/IgG ratio [5]. The utility of IgG4 for the diagnosis of autoimmune pancreatitis has been evaluated worldwide, with a sensitivity ranging from 50% to 92% and a specificity over 90%. Serum IgG4 concentration is therefore considered a reliable marker for the diagnosis of autoimmune pancreatitis and has been included in various diagnostic criteria [9–12]. Differences in sensitivity and specificity may be partly due to the use of different assays to measure serum IgG4 and different cut-offs for the upper limit of normal around the world, as well as variations in diagnostic criteria used in individual countries, which may be associated with histological differences between lymphoplasmacytic sclerosing pancreatitis (LPSP) [13] and idiopathic duct-centric chronic pancreatitis (IDCP) [14].

 Clinical features of autoimmune pancreatitis have been reported to differ based on the serum concentration of IgG4. Compared with patients having normal serum IgG4 levels, those with elevated IgG4 are regarded as being in a highly active state, with a higher incidence of jaundice at onset, more frequent diffuse pancreatic enlargement on imaging, significantly higher 18F-2-fluoro-2-deoxy-d-glucose uptake by pancreatic lesions, more frequent extrapancreatic lesions, and more frequent requirement for maintenance therapy [15]. 

 In addition, infiltration of IgG4 bearing plasma cells is a histological hallmark of autoimmune pancreatitis and is used in pathologic diagnoses [6]. 

## 3. IgG4 and the Differentiation of Autoimmune Pancreatitis from Pancreatic Cancer

 Lymphoplasmacytic sclerosing pancreatitis (LPSP), which is similar pathologically to autoimmune pancreatitis, has been observed in 2.5% of patients undergoing the Whipple resection [16]. Therefore, it is necessary to differentiate autoimmune pancreatitis from pancreatic cancer. We reported that IgG4 had a sensitivity of 90%, a specificity of 98%, and an accuracy of 95% in differentiating between these conditions, [5] indicating that IgG4 is useful both for the diagnosis of autoimmune pancreatitis and for differentiating it from pancreatic cancer. Other reports have also shown the usefulness of IgG4 in differential diagnosis [17–19]. The sensitivity and specificity of IgG4 were superior to those of IgG, ANA, and RF, although the additional measurement of ANA and RF further increased the sensitivity and negative predictive value of IgG4 [8].

## 4. IgG4 and Prediction of Relapse

 Some patients with autoimmune pancreatitis experience relapse during their clinical course. For effective management, it is necessary to determine the frequency of relapse and its prevention. During the period from 1992 to 2011, a total of 93 patients with autoimmune pancreatitis were examined and treated at Shinshu University Hospital. Of the 84 patients followed up for more than 1 year, 28 (33%) experienced relapse. In Japanese patients, the relapse rate has been estimated to vary from 30 to 50%, [20–22] although corticosteroid therapy significantly reduced relapse rates [22]. Japanese consensus guidelines for the management of autoimmune pancreatitis have stated that the indications for corticosteroid therapy include symptoms such as obstructive jaundice, abdominal pain, and back pain, and the presence of symptomatic extrapancreatic lesions. The major lesions at relapse included autoimmune pancreatitis (*n* = 26), sclerosing cholangitis (*n* = 18), lachrymal and salivary gland lesions (*n* = 5), and retroperitoneal fibrosis (*n* = 4). In addition, the involvement of other organs and symptoms were seen at relapse. We failed to identify any serum markers at diagnosis that could predict relapse, although we observed elevated concentrations of IgG and immune complex in the relapse compared with the nonrelapse group, although these differences were not significant. Serial changes in IgG4 and immune complexes in a 69-year-old woman with autoimmune pancreatitis who experienced 3 relapses showed that these markers were elevated in serum several months before clinically evident relapse, suggesting that regular measurements of these markers in an out-patient clinic may predict relapse [23].

## 5. Long-Term Followup of Patients with Autoimmune Pancreatitis and Normal or Elevated IgG4

 We followed 2 patients with autoimmune pancreatitis for 10 years each, a 55-year-old man with a serum IgG4 concentration of 1135 mg/dL and a 65-year-old woman with a serum IgG4 concentration of 42 mg/dL [24]. The first patient experienced several recurrences, developing a pancreatic stone and pancreatic duct stenosis, whereas the latter patient showed no duct changes over time. These findings suggest that autoimmune pancreatitis accompanied by normal IgG4 concentrations may represent lower activity and a nonprogressive state [24].

## 6. IgG4 and Extrapancreatic Lesions in Autoimmune Pancreatitis

 Extrapancreatic lesions in patients with autoimmune pancreatitis may involve organs throughout the entire body [7, 8]. Serum IgG4 concentrations were well correlated with the number of extrapancreatic organs involved, indicating a correlation between increased serum IgG4 and extrapancreatic involvement. Among the 5 types of extrapancreatic involvement, lachrymal and salivary gland lesions and hilar lymph adenopathy have been significantly associated with high serum IgG4 concentrations, suggesting that patients with high serum IgG4 should be assessed for the occurrence of these lesions [7]. However, a recent study of large numbers of patients with autoimmune pancreatitis and extrapancreatic lesions showed different results, as shown in Table 1. Patients with IgG4-related retroperitoneal fibrosis and kidney lesions had higher IgG4 concentrations than other patients, probably because these lesions were complications of many other IgG4-related diseases (Table 1).

## 7. Role of IgG4

 IgG4 in these patients may be (1) pathogenic, (2) anti-inflammatory, or (3) as a rheumatoid factor. For example, anti-desmoglein3 IgG4 autoantibody has been reported pathogenic for pemphigus vulgaris [25]. Transfer of an anti-desmoglein3 IgG4 autoantibody from a pemphigus vulgaris patient to BALB/C mice resulted in a pemphigus vulgaris like lesion, suggesting the involvement of an IgG4 autoantibody directed against an unknown target antigen. Similarly, IgG4 deposits have been detected in tissues of patients with autoimmune pancreatitis [26]. In contrast, IgG4 was found to have anti-inflammatory effects against allergic reactions. IgG4 antibodies can bind to soluble antigens, blocking the interaction between these antigens and IgE on mast cells and inhibiting allergic reactions. A dynamic Fab arm exchange of IgG4 can occur, resulting in bispecific activity, loss of monospecific cross-linking activity, and loss of the ability to form immune complexes, resulting in anti-inflammatory effects [27].

 IgG4 may act as an autoantibody against IgG or have rheumatoid factor activity. Western blotting has shown that IgG4 from the sera of patients with autoimmune pancreatitis can bind to IgG1, IgG2, IgG3, and IgG Fc [28]. Furthermore, IgG4 Fc, but not IgG4 Fab, was found to bind to IgG Fc [28], indicating that IgG4 binding to IgG Fc is via an Fc-Fc interaction, not via rheumatoid activity. ELISA showed that IgG4 from the serum of each patient with autoimmune pancreatitis could bind to IgG1 coated onto microplates, a binding well correlated with serum IgG4, but not rheumatoid factor, concentration [28]. The role of IgG4 Fc-IgG Fc is unclear, but it may have physiological and/or pathological effects, suggesting the need for further studies.

## 8. Conclusion

The utility of serum IgG4 concentration as a biomarker of the major IgG4-related disease, autoimmune pancreatitis, includes its ability to diagnose autoimmune pancreatitis as well as to differentiate this disease from pancreatic cancer. Moreover, serum IgG4 concentration may be a marker for predicting relapse and for the evaluation of extrapancreatic lesions. It remains unclear, however, whether IgG4 and its rheumatoid factor-like activity may be beneficial or pathogenic in these patients.

## Figures and Tables

**Table 1:** IgG4 concentrations, age and complications of more than 3 extrapancreatic lesions in patients with autoimmune pancreatitis and major extrapancreatic lesions.

	*n* (male/female)	Age, yr Median (range)	IgG4, mg/dL Median (range)	Complications of more than 3 extrapancreatic lesions, *n* (%)
Autoimmune pancreatitis	92 (72/20)	66 (38–85)	545 (4–2970)	11(12%)
Mikulicz's disease	41 (31/10)	65 (43–82)	697 (18–2970)	13 (32%)
Sclerosing cholangitis	71 (53/18)	67 (38–84)	500 (4–2970)	13 (18%)
Retroperitoneal fibrosis	27 (23/4)	66 (50–80)	1110 (247–2970)	12 (44%)
Kidney lesion	18 (14/4)	67 (56–82)	1313 (156–2970)	9 (50%)
